# Expression Status and Prognostic Value of m^6^A RNA Methylation Regulators in Lung Adenocarcinoma

**DOI:** 10.3390/life11070619

**Published:** 2021-06-26

**Authors:** Xiuhong Li, Zian Feng, Rui Wang, Jie Hu, Xiaodong He, Zuojun Shen

**Affiliations:** 1 Department of Clinical Laboratory, The First Affiliated Hospital of USTC, Division of Life Sciences and Medicine, University of Science and Technology of China, Hefei 230001, China; ls1997@mail.ustc.edu.cn (X.L.); fengzian@mail.ustc.edu.cn (Z.F.); hujie121@mail.ustc.edu.cn (J.H.); 2 Deparment of Clinical Laboratory, Anhui Provincial Hospital, Anhui Medical University, Hefei 230001, China; WangR4171@163.com; 3 Anhui Provincial Center for Clinical Laboratories, Hefei 230001, China; h_xd5431@sina.com

**Keywords:** m^6^A methylation, lung adenocarcinoma, prognostic signature, survival analysis

## Abstract

N^6^-methyladenosine (m^6^A) RNA modification is the most abundant modification method in mRNA, and it plays an important role in the occurrence and development of many cancers. This paper mainly discusses the role of m^6^A RNA methylation regulators in lung adenocarcinoma (LUAD) to identify novel prognostic biomarkers. The gene expression data of 19 m^6^A methylation regulators in LUAD patients and its relevant clinical parameters were extracted from The Cancer Genome Atlas (TCGA) database. We selected three significantly differentially expressed m^6^A regulators in LUAD to construct the risk signature, and evaluated its prognostic prediction efficiency using the receiver operating characteristic (ROC) curve. Kaplan–Meier survival analysis and Cox regression analysis were used to identify the independent prognostic significance of the risk signature. The ROC curve indicated that the area under the curve (AUC) was 0.659, which means that the risk signature had a good prediction efficiency. The results of the Kaplan–Meier survival analysis and Cox regression analysis showed that the risk score can be used as an independent prognostic factor for LUAD. In addition, we explored the differential signaling pathways and cellular processes related to m^6^A methylation regulators in LUAD.

## 1. Introduction

Lung cancer is the leading cause of cancer-related deaths worldwide [1]. There are many risk factors for lung cancer, with smoking and environmental and occupational exposure as the most common risk factors [2]. In the past few decades, medical technology has made great progress, but the treatment effect for lung cancer patients is not ideal. The five-year survival rate for lung cancer is reported to be 19%, one of the lowest five-year survival rates, while adenocarcinoma, the most common histological subtype of lung cancer, is more aggressive and has a poorer prognosis [3,4,5]. To relieve the current clinical treatment pressure and to improve the prognosis of patients, it is necessary to find reliable prognostic markers to optimize the treatment regimen for lung adenocarcinoma (LUAD).

N^6^-methyladenosine (m^6^A) is a methylated modification of RNA molecules that was first discovered in 1974 [6]. As of the end of 2017, more than 150 post-transcriptional modifications have been identified in all organisms, and m^6^A is the most common internal mRNA modification found in eukaryotes and plays a key role in a variety of basic biological processes such as cell differentiation, tissue development, and tumorigenesis [7,8,9,10]. In mammals, approximately 0.1–0.4% of adenosine in isolated RNA is m^6^A-modified, accounting for approximately 50% of the total methylated ribonucleotide [11]. Dominissini et al. [12] used a new method of antibi-mediated capture and massive parallel sequencing, m^6^A-seq, which found that m^6^A is clustered in the termination codon, the 3′ untranslated region (3′ UTR), and the internal exon. m^6^A methylation is a dynamic reversible process catalyzed by three types of proteases: Writers (methyltransferase complex, including METTL3/14/16, WTAP, IC3H13, ZC3H13, RBM15/15B, and KIAA1429), erasers (demethylases, including FTO and ALKBH5), and readers (including YTHDF1/2/3, IGF2BP1/2/3, YTHDC1/2, HNRNPC, HNRNPG, and HNRNPA2B1) [11,13,14]. Writers mediate the methylation modification process of RNA to “write” the methylation modification to RNA, and readers are responsible for “reading” the information of RNA methylation modification and participate in downstream RNA translation and degradation processes, and then rely on erasers mediating the process of RNA demethylation modification, which can “erase” the RNA methylation modification signal, thereby making the m^6^A modification process dynamic and reversible [15].

At present, many studies have shown that m^6^A methylation regulators are closely related to the occurrence and development of tumors. For example, Taketo et al. [16] indicated that METL3, as an m^6^A regulator, is up-regulated in patients with pancreatic cancer and is an effective target in the treatment of such patients. Maetal et al. [17] found that down-regulation of METTL14 expression is a poor prognostic factor for hepatocellular carcinoma and is closely related to tumor metastasis. However, there is still insufficient information about the role of m^6^A RNA methylation regulators in LUAD. Therefore, in this study, RNA sequencing data were obtained from The Cancer Genome Atlas (TCGA), and the expression data of 19 m^6^A methylation regulators in 535 LUAD tumor tissue samples and 59 normal tissue samples were systematically analyzed, as well as their association with clinicopathological characteristics. We used the least absolute shrinkage and selection operator (LASSO) Cox regression algorithm to analyze 19 m^6^A methylation regulators, and selected IGF2BP1, HNRNPC, and HNRNPA2B1 to construct the minimum standard risk signature; meanwhile, Kaplan–Meier survival analysis and univariate and multivariate Cox regression analyses were used to identify the predictive effect of the risk signature on the prognosis of LUAD patients. Gene Set Enrichment Analysis (GSEA), Gene Ontology (GO), and Kyoto Encyclopedia of Genes and Genomes (KEGG) analyses were used for further functional annotation.

## 2. Materials and Methods

### 2.1. Data Acquisition

All data in this study were downloaded from the TCGA (https://cancergenome.nih.gov/, accessed on 30 December 2020) database, including gene expression data and the corresponding clinical information of 535 LUAD tumor tissue samples and 59 normal tissue samples. If any parameter value was missing, the entire patient data were excluded from the analysis. After screening, the clinical data of 479 samples were retained (Supplementary Table S1). The clinical–demographic features of the patients with LUAD are detailed in Table 1.

### 2.2. Selection of m^6^A Methylation Regulators and Analysis of Their Differential Expression

The TCGA database provides the expression data of 19 m^6^A methylation regulators, i.e., YTHDF3, YTHDF2, YTHDF1, KIAA1429, HNRNPA2B1, RBM15, METTL3, HNRNPC, IGF2BP2, IGF2BP3, IGF2BP1, FTO, ZC3H13, WTAP, METTL14, ALKBH3, ALKBH5, YTHDC1, and YTHDC2. In order to identify the expression of m^6^A RNA methylation regulators in LUAD, the Limma package [18] was used to analyze the expression of 19 m^6^A RNA methylation regulators in 479 LUAD tumor tissues and 59 normal tissues, and the expression levels of 19 m^6^A RNA methylation regulators in LUAD tumor tissue samples with different clinical characteristics were compared, and the expression levels were evaluated by *t*-tests. Utilizing the pheatmap package, the results were used to generate a heatmap and a vioplot for visualization purposes.

### 2.3. Correlation Analysis of m^6^A Methylation Regulators

In order to further study the correlation between m^6^A methylation regulators, co-expression correlation analysis was carried out, and the results were visualized by the “corrplot” package. We performed univariate Cox regression analysis on the expression of 19 m^6^A RNA methylation regulators in 479 LUAD tumor tissues, and genes with *p* < 0.05 were considered to be significantly associated with the survival of LUAD patients.

### 2.4. Construction and Verification of Risk Signature

To verify the prognostic effect of m^6^A RNA methylation regulators in LUAD patients, we performed LASSO Cox regression analysis [19,20] on 15 m^6^A RNA methylation regulators significantly related to the survival of patients, and screened three m^6^A RNA methylation regulators (IGF2BP1, HNRNPC, and HNRNPA2B1) to construct a minimum standard risk signature (Supplementary Figure S1a,b) The obtained coefficients (IGF2BP1 coefficient = 0.0352, HNRNPC coefficient = 0.0046, and HNRNPA2B1 coefficient = 0.0006) were used to calculate the risk score of the TCGA dataset. The risk score calculation formula is as follows (Coef_i_ means coefficient and Exp_i_ means the expression value of each selected gene):

(1) $$\mathrm{Risk}\;\mathrm{score}\;=\overset{n}{\underset{i\;=1}{\sum }}{\mathrm{Coef}}_{i}\times {\mathrm{Exp}}_{i}$$

Additionally, the patients were classified into low- and high-risk groups according to the median of the risk scores. Principal component analysis (PCA) was performed on the grouping results by the Limma package, and the results were visualized by the ggplot2 package. The survival package [21] was used to compare the overall survival (OS) rate of the high- and low-risk groups by the Kaplan–Meier method. Then, we constructed a receiver operating characteristic (ROC) curve [22] to evaluate the prediction efficiency of the risk signature.

### 2.5. Analysis of the Prognostic Ability of the Three-Gene Signature

The “pheatmap” package was used to generate heatmaps to visually analyze the expression differences of the three genes in the high- and low-risk groups, as well as the expression differences of the three genes in LUAD patients with different clinicopathological characteristics. Univariate and multivariate independent prognostic analyses of the risk scores were performed to identify the prognostic value of the risk signature. GSEA was used to annotate the differential signaling pathways and cellular processes between the two groups. GO [23] enrichment and KEGG [24] pathway analysis were used to analyze the differentially expressed genes (DEGs) between the high- and low-risk groups. When the *p*-value was less than 0.05, the enrichment pathway was considered to be statistically significant.

### 2.6. Statistical Analysis

One-way analysis of variance was used to compare the expression of m^6^A RNA methylation regulators in the tumor tissues of TCGA LUAD patients. The relationship between m^6^A RNA methylation regulators and the clinicopathological characteristics of LUAD patients was analyzed by *t*-tests. OS was defined as the time interval from the date of diagnosis to the date of death. The Kaplan–Meier method was used for OS analysis to conduct a bilateral log-rank test. All statistical analyses were performed using R software (version 3.6.2), and *p* < 0.05 was considered statistically significant.

## 3. Results

### 3.1. Expression of m^6^A RNA Methylation Regulators in LUAD

**Figure 1:**
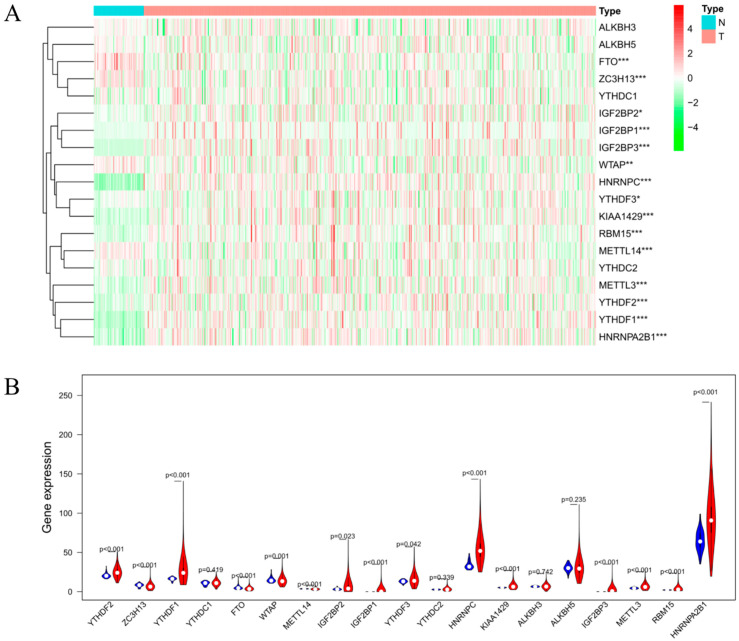
Bioinformatics analysis of the expression of m^6^A RNA methylation regulators in LUAD. (**A**) Heatmap of the expression of m^6^A RNA methylation regulator in normal tissues (N, blue) and LUAD tumor tissues (T, pink). Red represents up-regulation and green represents down-regulation. (**B**) Vioplot visualizing the differentially expressed m^6^A RNA methylation regulators in LUAD. * *p* < 0.05, ** *p* < 0.01, and *** *p* < 0.001.

We compared the expression levels of 19 m^6^A RNA methylation regulators in 535 LUAD tumor tissue samples and 59 normal tissue samples extracted from the TCGA database. As shown in Figure 1A,B, we found that there was a significant difference in the expression levels of YTHDF3, YTHDF2, YTHDF1, KIAA1429, HNRNPA2B1, RBM15, METTL3, HNRNPC, IGF2BP2, IGF2BP3, IGF2BP1, FTO, ZC3H13, WTAP, and METTL14 between LUAD tumor tissues and normal tissues. Among these regulators, the expression levels of YTHDF3, YTHDF2, KIAA1429, HNRNPA2B1, RBM15, METTL3, HNRNPC, YTHDF1, IGF2BP2, IGF2BP3, and IGF2BP1 in LUAD tumor tissues were significantly higher than those in normal tissues, while FTO, ZC3H13, WTAP, and METTL14 were lower than in normal tissues.

### 3.2. Correlation among the 19 m^6^A RNA Methylation Regulators in LUAD

**Figure 2:**
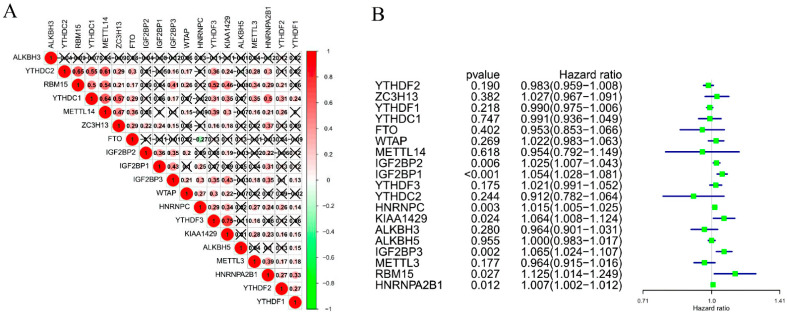
The correlation among the 19 selected m^6^A RNA methylation regulators. (**A**) Spearman correlation analysis of m^6^A RNA methylation regulators in LUAD. (**B**) The hazard ratio (HR) and 95% confidence interval (CI) of m^6^A RNA methylation regulators calculated by univariate COX regression analysis.

To further understand the correlation among the 19 m^6^A RNA methylation regulators in LUAD, we analyzed the correlation of 19 m^6^A RNA methylation regulators. The results are shown in Figure 2A, highlighting obvious correlations among the 19 m^6^A RNA methylation regulators, most of which were positive correlations. Among them, YTHDC2 and RBM15, YTHDC1 and METTL14, and YTHDC2, and METL14 demonstrated the strongest positive correlations.

We performed univariate Cox regression analysis on 19 m^6^A methylation regulators to identify the regulators in the LUAD dataset associated with the survival of LUAD patients. As shown in Figure 2B, the expression of IGF2BP1 (HR = 1.054, 95%CI = 1.028–1.081), IGF2BP2 (HR = 1.025, 95%CI = 1.007–1.043), IGF2BP3 (HR = 1.065, 95%CI = 1.024–1.107), HNRNPC (HR = 1.015, 95%CI = 1.005–1.025), RBM15 (HR = 1.125, 95%CI = 1.014–1.249), HNRNPA2B1 (HR = 1.007, 95%CI = 1.002–1.012), and KIAA1429 (HR = 1.064, 95%CI = 1.008–1.124) was significantly associated with the survival of LUAD patients, while IGF2BP1 (*p* < 0.001) was the most related to the survival of LUAD patients.

### 3.3. Evaluation of the m^6^A-Related Risk Signature

**Figure 3:**
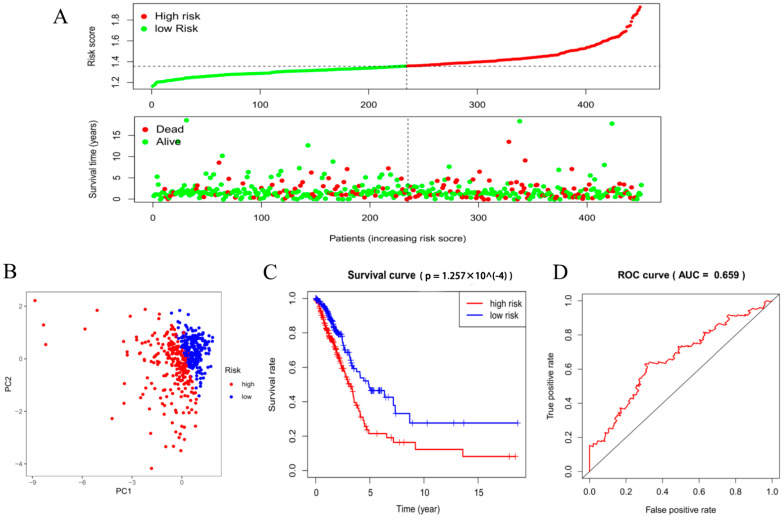
Analysis of the survival risk signature for LUAD patients. (**A**) The risk score curve of each sample in the LUAD dataset of the TCGA database. (**B**) PCA based on the median grouping of risk scores. (**C**) Kaplan–Meier survival analysis curve of the high- and low-risk groups. (**D**) The constructed ROC curve evaluates the predictive efficiency of the risk signature for the survival rate of LUAD patients, AUC = 0.659.

To explore the prognostic value of the three-gene risk signature, we divided the LUAD patients obtained from TCGA into a low- and a high-risk group based on the median risk score. As shown in Figure 3A, as the risk score increased, the number of deaths in the high-risk group became significantly higher than in the low-risk group. We also performed PCA on the risk signature to compare the differences between the two groups. The results showed that the distribution directions of the two groups were different and there was a clear boundary, suggesting that the risk signature could divide LUAD patients into two groups (Figure 3B). Then, we constructed a Kaplan–Meier survival curve to analyze the OS rate of the two groups. The results are shown in Figure 3C; there was a significant difference in the OS rate between the high- and low-risk groups (*p* = 1.257 × 10^−4^). The OS rate of the LUAD patients in the low-risk group was significantly higher than that of the LUAD patients in the high-risk group. In the follow-up, an ROC curve was established to evaluate the efficiency of the risk signature for predicting the five-year survival rate of LUAD patients. As shown in Figure 3D, the AUC was 0.659, indicating that the risk signature had a good predictive efficiency on the five-year survival rate of LUAD patients.

### 3.4. Prognostic Analysis of the m^6^A-Related Risk Signature

**Figure 4:**
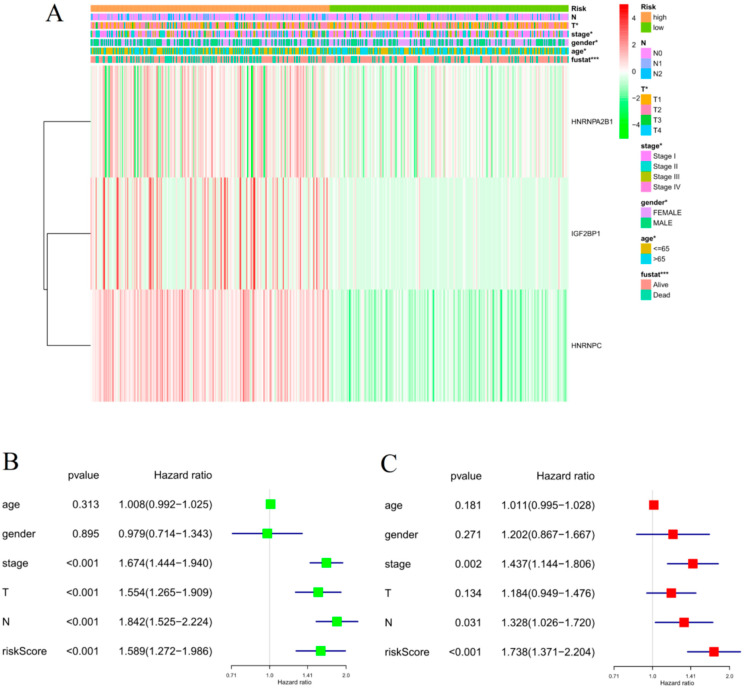
The relationship between the risk score and clinicopathological characteristics of LUAD. (**A**) Heatmap of the expression of three m^6^A RNA methylation regulators and the distribution of clinicopathological variables between the high- and low-risk groups. * *p* < 0.05, ** *p* < 0.01, and *** *p* < 0.001. (**B**,**C**) Univariate and multivariate independent prognostic analyses among the clinicopathological characteristics, risk scores, and OS of patients. The horizontal line in the color module represents the confidence interval of each factor.

After dividing the patients in a high- and a low-risk group according to the median risk score, we further compared the clinicopathological characteristics and the expression of three genes between the two groups. The heatmap in Figure 4A shows that there were significant differences at T, stage, and status between the high- and low-risk groups, and the expressions of IGF2BP1, HNRNPC, and HNRNPA2B1 in the high-risk group were up-regulated, while those in the low-risk group were down-regulated.

To verify whether the risk signature could be used as an independent prognostic indicator of LUAD, we conducted univariate and multivariate independent prognostic analyses on the risk score. As shown in Figure 4B,C, the results of the univariate Cox independent prognostic analysis showed that risk score (*p* < 0.001), stage (*p* < 0.001), T (*p* < 0.001), and N (*p* < 0.001) were significantly related to OS in LUAD patients. The results of the multivariate Cox independent prognosis analysis showed risk score (*p* < 0.001), stage (*p* = 0.002), and N (*p* = 0.031), which means that the risk signature could be used as an independent prognostic factor for LUAD.

### 3.5. Functional Enrichment Analysis

**Figure 5:**
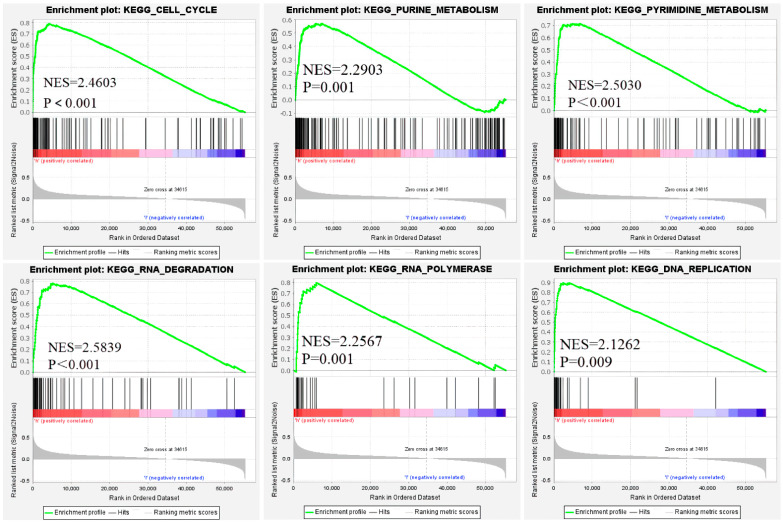
Function enrichment analysis based on the risk signature by GSEA. The genes involved in the six functional pathways in the figure were significantly up-regulated in the LUAD high-risk group.

We used GSEA to analyze the active signal pathways in the high- and the low-risk groups. There were dramatic differences in the expression of genes involved in the active pathways of the two groups. For example, the genes involved in the cell cycle, genetic material (purine and pyrimidine) metabolism, DNA replication, and translation process (RNA polymerase and RNA degradation) were significantly up-regulated in the high-risk group (Figure 5). Ayelet Erez et al. found that in many cancerous tumors, nitrogen is used to synthesize pyrimidines, which in turn supports the synthesis of RNA and DNA in cancer cells, disrupts the balance of the cell cycle, and promotes cancer progression.

**Figure 6:**
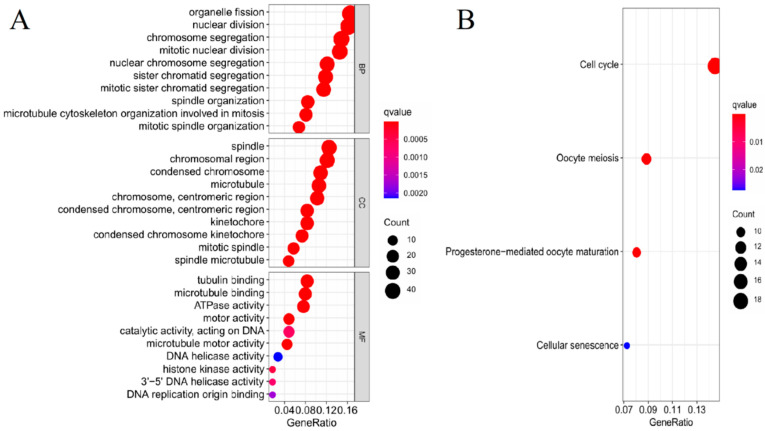
GO and KEGG analysis of the DEGs between the high- and low-risk groups. (**A**,**B**) The most significant GO enrichment and KEGG pathway in the DEGs between the high- and low-risk groups. The red depth represents greater significance, the blue depth represents lower significance, and the bubble size represents the number of genes.

After GESA analysis, we screened out a total of 378 DEGs (Log FC > 1, *p* < 0.05) between the high- and low-risk groups. To future explore the biological functions and pathways that had certain correlation with risk score, we performed GO enrichment and KEGG pathway analysis on the screened DEGs. The GO analysis results showed that the DEGs were mainly enriched in the biological processes associated with cell proliferation, including organelle fission, nuclear division, and chromosome segregation. Simultaneously, DEGs were enriched in cell components associated with mitosis, such as spindle and condensed chromosomes. There was also a significant enrichment in DEGs in the molecular functions associated with mitosis, covering tubulin binding, microtubule binding, and ATPase activity, while the KEGG analysis results showed that DEGs are mainly enriched in the cell cycle, which are likely to be associated with proliferation and metastasis during tumor progression (Figure 6A,B).

## 4. Discussion

LUAD is the most common subtype of non-small cell lung cancer, and its incidence is increasing year on year, accounting for almost 50% of all lung cancers. LUAD has a lower OS than most cancers due to its rapid development and invasiveness [5]. Traditional treatment methods include surgery, radiotherapy, and chemotherapy [25], and the choice of treatment method depends on the type of cancer (small cell or non-small cell), stage of development, and genetic characteristics [26]. If it can be detected at an early stage, surgical removal of non-small cell lung cancer can provide a good prognosis. However, more than 70% of patients with non-small cell lung cancer are diagnosed with advanced or metastatic disease, which leads to a poor prognosis and low five-year survival rate [27]. Therefore, there is an urgent need to explore potential prognostic biomarkers and promising targets, such as the prognostic value and prediction prospects of cellular and molecular immune markers in lung cancer [28], which can be used to develop an appropriate treatment plan for patients to prolong their survival time.

As the most common modification in human mRNA, m^6^A methylation modification is involved in regulating mRNA processing, translation, and stability [29]. Abnormal regulation of m^6^A modification plays an important role in many types of tumors by affecting the expression of tumor-related genes. Many studies have confirmed that m^6^A modification is related to tumor proliferation, differentiation, tumorigenesis, invasion, and metastasis [17,30,31]. The levels of METTL3 and METTL14 in AML show significant changes, which affects the proliferation of AML cells and the process of tumor progression [32,33]. Studies have shown that METL3 and YTHDF1 levels are higher in liver cancer and are associated with a poor OS rate [34]. METTL3 can promote the expression of multiple oncogenes such as BRD4, EGFR, TAZ, MAPKAPK2, and DNMT3A in human lung cancer cells [35]. However, the influence of m^6^A methylation regulators on the prognosis of patients with LUAD needs to be further clarified. Therefore, in this study, we explored the value of m^6^A RNA methylation regulators in the prognosis of patients with LUAD.

Different m^6^A RNA methylation regulators have different effects on the same cancer, and they can play a role in promoting and inhibiting, the occurrence and development of tumors. On the one hand, the target gene modified by m^6^A can be either an oncogene or a tumor suppressor gene; on the other hand, the modified m^6^A can affect its target mRNA by recruiting different “readers” to play different roles in tumorigenesis and development. The same m^6^A methylation regulator is expressed differently in different cancers; for example, as an “eraser” in the process of m^6^A RNA methylation, FTO is highly expressed in breast, liver, and gastric cancer tissues compared to normal tissues, which is associated with poor prognosis, while FTO expression in bladder cancer tissues is lower than that in normal tissues [35,36,37,38]. Interestingly, in this study, we analyzed the data of 479 LUAD patients extracted from the TCGA database and showed that the 19 m^6^A RNA methylation regulators we studied had abnormal expression in LUAD patients. Among them, the expression of FTO in the tumor tissues of LUAD patients is significantly lower than that in normal tissues, which further validates the above viewpoint.

Insulin-like growth factor 2 mRNA-binding proteins (IGF2BPs; IGF2BP1/2/3), as a newly discovered m^6^A regulator in recent years, promotes the stability and storage of its target mRNA (such as MYC) in an m^6^A-dependent manner, and has a carcinogenic effect in cancer [39,40]. More and more studies have found that IGF2BP1 is abnormally expressed in liver cancer, lung cancer, colon cancer, ovarian cancer, breast cancer, and other tumors [41,42,43,44]. IGF2BP1 is not only related to the proliferation, migration, and invasion of tumor cells, but also closely related to the poor prognosis of patients [45]. Interestingly, we performed univariate Cox regression analysis on 19 m^6^A regulatory factors and found that IGF2BPs were significantly related to the survival of LUAD patients, and were significantly highly expressed in the tumor tissues of LUAD patients. Therefore, we believe that the high expression of IGF2BPs may be one of the factors leading to the occurrence and development of LUAD in patients, and further study of the mechanism of IGF2BP1 in malignant tumors may be expected to provide a new method for tumor-targeted therapy. Xu et al. found that the YTHDC2–IGF2BP2–HNRNPC risk prognosis model has important application value in the prognosis assessment of oral cancer, which may be related to remodeling of the tumor-related immune microenvironment. Yang et al. found that hnRNPA2/B1’s, a new COX-2 modulator, up-regulated expression predicts poor prognosis for NSCLC patients, indicating that hnRNPA2/B1 promotes tumors [46].

Next, based on the abovementioned research and our findings, we selected three m^6^A RNA methylation regulators (IGF2BP1, HNRNPC, and HNRNPA2B1) to construct a minimum standard risk signature, which had a good predictive effect on the prognosis of LUAD patients. The higher the signature-based risk score, the worse the prognosis. At the same time, we divided 479 LUAD patients into high- and low-risk groups based on the median risk score and verified the results with PCA, showing that the results of grouping according to the median risk score have a clear boundary. The Kaplan–Meier survival analysis results showed that the signature can significantly distinguish LUAD patients with different OS, and also has a good predictive efficiency on the prognosis of LUAD patients (AUC = 0.659). Moreover, we also performed univariate and multivariate Cox independent prognostic analyses of the risk score, and the results showed that the risk score is an independent prognostic factor of LUAD. The results of the GESA analysis showed that the poor prognosis of the high-risk group may be related to the active cell cycle, genetic material metabolism, gene replication, and translation process, which may promote tumor progression. The results of the GO and KEGG analysis of the DEGs between the two groups suggested that DEGs were also mainly enriched in the cell cycle process, especially mitosis, and may affect the progress of LUAD through the regulation of mitosis. Since the grouping of patients relied on three m^6^A methylation regulators, this implies that they may directly or indirectly regulate the mRNA expression of DEGs between the two groups, thereby affecting the prognosis of patients through the regulation of cell cycle and mitosis These results may provide a theoretical basis for further research on the pathogenesis of LUAD and the establishment of new risk classification and prognostic models.

## 5. Conclusions

In summary, our research established a novel prognostic risk signature on the basis of three m^6^A RNA methylation regulators (IGF2BP1, HNRNPC, and HNRNPA2B1). Additionally, the risk signature was verified to be an independent prognostic factor of LUAD, which provides a new direction for the prognosis prediction of LUAD. However, our study still has some limitations. First, the risk signature was developed using the TCGA cohort, and the predictive effectiveness of the signature needs to be validated in other prospective cohorts. Second, the underlying mechanisms between the DEGs in the high- and low-risk groups and cell cycle, especially mitosis, have to be studied in the future. Otherwise, the specific role of IGF2BP1, HNRNPC, and HNRNPA2B1 in LUAD needs further experimental exploration.

## Figures and Tables

**Table 1:** Clinical–demographic features of patients with LUAD.

Feature	*N* (479)	Rate %
Age (years)		
>65	251	52.4%
≤65	228	47.6%
gender		
Female	256	53.4%
Male	223	46.6%
T classification		
T1	165	34.5%
T2	254	53.0%
T3	44	9.2%
T4	16	3.3%
N classification		
N0	317	66.2%
N1	93	19.4%
N2	69	14.4%
TNM stage		
I	260	54.2%
II	120	25.1%
III	78	16.3%
IV	21	4.4%

## Data Availability

Additional data not presented in the manuscript can be obtained by contacting the authors.

## Supplementary Materials

The following are available online at https://www.mdpi.com/article/10.3390/life11070619/s1, Table S1: 479 LUAD Patients information. Figure S1: Construction of a 10-gene signature model in the TCGA cohort.

## Abbreviations

m^6^A, N^6^-methyladenosine; LUAD, lung adenocarcinoma; TCGA, The Cancer Genome Atlas; OS, overall survival; ROC, receiver operating characteristic; KEGG, Kyoto Encyclopedia of Genes and Gene; GSEA, Gene Set Enrichment Analysis; GO, Gene Ontology; DEGs, differentially expressed genes.
