# Supplementary material for: Expression Status and Prognostic Value of m6A RNA Methylation Regulators in Lung Adenocarcinoma
Source: Life (Basel). 2021 Jun 26;11(7):619. doi: 10.3390/life11070619 (PMC8306618; doi:10.3390/life11070619)
Supplement: Supplementary file 1 [file life-11-00619-s001.zip › life-1174247-supplementary/Supplemental material 1.pdf]

Table S1. 479 LUAD Patients information

id	survival time (month)	survival status
TCGA-L4-A4E5	19.27	0
TCGA-50-6673	0.73	1
TCGA-35-4123	6.07	0
TCGA-86-8076	16.30	0
TCGA-NJ-A4YF	72.03	0
TCGA-55-6980	70.30	0
TCGA-83-5908	27.47	0
TCGA-44-3918	6.57	0
TCGA-50-7109	10.27	1
TCGA-64-1681	38.90	1
TCGA-44-6145	10.93	0
TCGA-MP-A4TJ	11.30	1
TCGA-44-2655	19.17	0
TCGA-73-4662	30.40	0
TCGA-44-2664	12.77	0
TCGA-L4-A4E6	14.50	0
TCGA-50-5942	29.43	0
TCGA-35-4122	7.50	0
TCGA-44-2661	14.87	0
TCGA-50-5944	25.47	0
TCGA-67-3770	20.33	0
TCGA-05-4389	45.63	0
TCGA-44-3919	6.33	0
TCGA-MP-A4T2	37.87	1
TCGA-38-7271	26.67	1
TCGA-49-4487	28.50	1
TCGA-49-4486	77.27	1
TCGA-44-A47G	11.70	0
TCGA-55-1595	49.30	0
TCGA-MP-A4TA	31.67	1
TCGA-78-7162	105.63	1
TCGA-67-3771	20.33	0
TCGA-55-6987	71.23	0
TCGA-78-8655	78.67	0
TCGA-49-4514	56.67	0
TCGA-91-6835	2.63	0
TCGA-50-5935	21.77	1
TCGA-97-A4M6	18.93	0
TCGA-99-8028	37.27	0
TCGA-97-A4M1	20.03	0
TCGA-55-8089	23.40	1
TCGA-67-6216	4.70	0

TCGA-44-6144	13.57	0
TCGA-64-1676	57.60	0
TCGA-99-8032	1.47	0
TCGA-MN-A4N5	2.80	0
TCGA-50-8457	25.97	0
TCGA-97-A4M2	20.80	0
TCGA-95-7947	1.33	0
TCGA-91-6828	10.77	0
TCGA-MP-A4TH	14.87	0
TCGA-99-AA5R	21.93	0
TCGA-95-7944	0.70	0
TCGA-69-8255	4.30	0
TCGA-97-8179	14.50	0
TCGA-50-8460	27.63	0
TCGA-86-A456	13.50	0
TCGA-55-8090	19.93	1
TCGA-69-7980	13.70	0
TCGA-4B-A93V	8.47	0
TCGA-44-7672	13.93	0
TCGA-55-8206	29.60	0
TCGA-MN-A4N4	39.17	0
TCGA-73-7498	39.63	0
TCGA-91-6840	12.40	0
TCGA-44-6148	12.07	0
TCGA-86-8668	14.10	0
TCGA-55-8085	30.13	0
TCGA-86-8669	31.27	0
TCGA-S2-AA1A	17.10	0
TCGA-97-A4M3	18.00	0
TCGA-55-8203	18.23	0
TCGA-86-7953	19.70	0
TCGA-69-7763	23.00	0
TCGA-NJ-A4YQ	29.53	0
TCGA-55-7281	29.07	0
TCGA-44-A4SS	13.83	0
TCGA-55-8208	22.47	0
TCGA-55-7995	29.63	0
TCGA-97-A4M7	20.97	0
TCGA-69-7764	13.80	0
TCGA-44-3398	8.43	0
TCGA-55-A57B	18.20	0
TCGA-97-A4M5	21.13	0
TCGA-55-1592	23.37	1
TCGA-50-5049	71.53	0

TCGA-44-3917	13.07	0
TCGA-05-4420	30.40	0
TCGA-44-2666	3.23	1
TCGA-78-7535	31.63	1
TCGA-71-6725	8.53	0
TCGA-44-2668	8.20	0
TCGA-05-4417	15.17	0
TCGA-55-6969	41.30	0
TCGA-78-8662	112.03	1
TCGA-62-A46R	57.50	1
TCGA-64-5781	40.07	0
TCGA-78-7542	10.70	1
TCGA-35-3615	0.47	0
TCGA-05-4390	37.53	0
TCGA-44-2656	19.40	0
TCGA-05-4430	25.37	0
TCGA-64-5778	30.87	0
TCGA-64-5774	85.00	0
TCGA-78-7163	227.07	0
TCGA-71-8520	7.00	1
TCGA-86-8673	15.17	0
TCGA-78-7143	165.37	1
TCGA-44-2662	16.00	0
TCGA-78-7153	25.33	0
TCGA-78-7152	40.07	0
TCGA-NJ-A4YG	63.47	0
TCGA-62-A46P	19.80	1
TCGA-62-A46O	48.47	1
TCGA-86-8075	15.97	0
TCGA-78-7540	29.37	0
TCGA-78-7633	33.13	0
TCGA-05-4249	50.77	0
TCGA-49-4501	47.37	1
TCGA-MP-A4SV	87.33	1
TCGA-05-4422	12.17	0
TCGA-86-7954	20.17	0
TCGA-05-4382	20.23	0
TCGA-78-7155	39.03	1
TCGA-05-4426	26.37	0
TCGA-50-6592	25.90	1
TCGA-50-5066	31.47	0
TCGA-38-4631	11.80	1
TCGA-50-6590	42.93	1
TCGA-78-7537	54.07	1

TCGA-55-6972	54.40	1
TCGA-67-3774	12.83	0
TCGA-62-A46S	55.10	1
TCGA-05-4405	20.33	0
TCGA-55-6986	108.70	0
TCGA-50-5931	14.47	1
TCGA-38-4630	35.77	1
TCGA-NJ-A55A	0.27	0
TCGA-05-4403	19.27	0
TCGA-MP-A5C7	62.07	0
TCGA-62-A46V	73.30	0
TCGA-50-6597	42.27	1
TCGA-73-4658	53.33	1
TCGA-67-3772	19.10	0
TCGA-05-4433	24.33	0
TCGA-67-3773	14.23	0
TCGA-62-A470	39.80	1
TCGA-50-5939	15.33	1
TCGA-95-7948	4.43	0
TCGA-69-7973	7.67	0
TCGA-86-8358	21.77	0
TCGA-NJ-A4YP	1.67	0
TCGA-67-6215	5.80	0
TCGA-55-A493	0.93	0
TCGA-44-8117	12.83	0
TCGA-55-7576	22.33	0
TCGA-97-8175	18.37	0
TCGA-86-8585	11.77	0
TCGA-55-8616	1.60	0
TCGA-44-8120	8.67	0
TCGA-86-8073	24.67	0
TCGA-97-8177	16.63	0
TCGA-44-4112	12.33	0
TCGA-97-A4M0	21.73	0
TCGA-86-7955	16.93	0
TCGA-86-A4P7	13.83	0
TCGA-44-7671	17.83	0
TCGA-55-7574	33.17	1
TCGA-38-4625	99.10	0
TCGA-05-5715	2.07	0
TCGA-44-7661	12.20	0
TCGA-97-8172	18.17	0
TCGA-44-A47B	9.57	0
TCGA-38-A44F	4.43	0

TCGA-97-A4LX	20.47	0
TCGA-73-7499	23.83	0
TCGA-86-A4D0	3.87	1
TCGA-55-7910	34.67	0
TCGA-86-8280	23.37	0
TCGA-MP-A4TF	11.20	1
TCGA-97-8174	5.47	1
TCGA-55-A48Y	21.00	0
TCGA-86-7713	14.07	0
TCGA-78-7539	10.90	0
TCGA-55-8205	19.97	0
TCGA-73-A9RS	11.33	1
TCGA-86-A4JF	17.87	0
TCGA-78-8648	40.30	1
TCGA-86-8672	0.63	1
TCGA-69-A59K	19.70	0
TCGA-44-6146	10.07	0
TCGA-50-8459	19.63	0
TCGA-38-4629	28.80	1
TCGA-78-7161	7.17	0
TCGA-62-A472	30.33	0
TCGA-05-4424	30.43	0
TCGA-62-8397	42.97	0
TCGA-69-7760	6.73	0
TCGA-44-8119	9.50	0
TCGA-62-8395	40.53	0
TCGA-86-8056	4.63	0
TCGA-64-5775	2.07	1
TCGA-99-7458	13.07	0
TCGA-53-7813	14.13	0
TCGA-78-7149	36.43	0
TCGA-73-4666	26.67	0
TCGA-55-6968	43.10	1
TCGA-53-7624	34.77	1
TCGA-50-6591	3.97	1
TCGA-86-7701	15.80	0
TCGA-73-4670	4.37	0
TCGA-05-4425	22.30	0
TCGA-MP-A4T7	5.57	1
TCGA-78-7167	89.37	1
TCGA-93-A4JN	23.93	0
TCGA-55-8094	18.03	0
TCGA-49-AARE	40.97	1
TCGA-49-AARN	37.83	1

TCGA-49-AAR0	155.17	0
TCGA-97-7553	62.33	0
TCGA-44-6778	37.00	0
TCGA-44-6776	64.60	0
TCGA-50-5946	22.87	0
TCGA-49-AARR	166.40	0
TCGA-95-8039	0.90	0
TCGA-49-4488	28.97	1
TCGA-O1-A52J	59.93	1
TCGA-97-7546	42.83	0
TCGA-97-8552	20.87	0
TCGA-49-AARO	125.30	0
TCGA-55-8507	13.93	0
TCGA-L9-A444	10.23	0
TCGA-55-8097	15.87	0
TCGA-55-7570	27.47	0
TCGA-J2-A4AD	18.33	1
TCGA-95-7043	0.07	0
TCGA-L9-A443	6.43	1
TCGA-44-A4SU	13.63	1
TCGA-55-7725	14.73	0
TCGA-55-7911	17.90	0
TCGA-55-A492	19.87	0
TCGA-93-A4JO	1.10	1
TCGA-93-7348	4.23	0
TCGA-55-8621	17.17	0
TCGA-91-8497	14.47	1
TCGA-93-7347	9.90	0
TCGA-97-7938	0.60	1
TCGA-J2-A4AE	22.37	0
TCGA-93-A4JQ	17.53	0
TCGA-55-6543	14.50	0
TCGA-55-8299	15.63	1
TCGA-55-7913	18.70	1
TCGA-55-7903	18.90	0
TCGA-J2-A4AG	16.67	0
TCGA-NJ-A55R	20.10	0
TCGA-44-7659	14.80	0
TCGA-55-A4DG	20.27	0
TCGA-97-7941	16.13	0
TCGA-55-7573	16.23	0
TCGA-55-7726	21.73	0
TCGA-91-8499	1.20	0
TCGA-55-A491	20.87	0

TCGA-55-A4DF	14.67	1
TCGA-49-AARQ	224.40	0
TCGA-91-6836	13.90	0
TCGA-55-8302	15.93	0
TCGA-55-6985	41.10	0
TCGA-55-6971	46.67	0
TCGA-91-6847	28.07	0
TCGA-55-6642	81.63	0
TCGA-49-AAR2	74.13	0
TCGA-91-6831	10.33	0
TCGA-97-7547	65.50	0
TCGA-97-7552	64.40	0
TCGA-44-7660	10.83	0
TCGA-44-A479	13.07	0
TCGA-55-8091	20.00	0
TCGA-91-6829	41.93	1
TCGA-55-8510	17.97	0
TCGA-55-8301	17.80	0
TCGA-55-8087	15.40	0
TCGA-44-7662	7.27	0
TCGA-55-A494	16.03	0
TCGA-L9-A8F4	15.87	0
TCGA-55-7728	23.47	0
TCGA-97-7937	18.80	0
TCGA-55-8096	23.97	1
TCGA-55-8514	17.33	0
TCGA-69-7979	2.97	0
TCGA-44-6775	12.33	0
TCGA-55-8511	18.40	0
TCGA-55-8207	32.57	0
TCGA-55-8614	17.87	0
TCGA-55-7724	23.50	0
TCGA-55-7815	25.77	0
TCGA-93-8067	6.20	0
TCGA-44-A47A	15.53	0
TCGA-69-7761	6.20	0
TCGA-55-8204	17.17	0
TCGA-MP-A4TE	29.87	1
TCGA-91-A4BC	1.47	0
TCGA-55-A490	3.30	1
TCGA-55-6978	5.87	1
TCGA-49-6767	22.57	0
TCGA-44-7667	18.57	0
TCGA-95-7039	1.13	0

TCGA-49-AAR9	8.67	1
TCGA-55-8506	0.37	0
TCGA-91-7771	16.40	0
TCGA-J2-8194	24.13	0
TCGA-55-8619	13.87	0
TCGA-55-7284	8.10	1
TCGA-55-8092	5.13	1
TCGA-69-8453	18.33	0
TCGA-55-8513	26.37	0
TCGA-55-7994	20.10	0
TCGA-44-5644	16.60	0
TCGA-78-8640	217.60	0
TCGA-53-7626	30.97	1
TCGA-50-5055	61.00	1
TCGA-44-2659	17.63	0
TCGA-44-7670	17.70	0
TCGA-NJ-A55O	0.43	0
TCGA-05-5428	22.33	0
TCGA-86-8074	0.80	0
TCGA-55-A48X	22.97	0
TCGA-38-4627	38.23	1
TCGA-86-6851	5.97	0
TCGA-49-4510	29.87	1
TCGA-MP-A4SW	59.27	1
TCGA-44-2665	13.33	0
TCGA-55-1596	68.83	0
TCGA-67-3776	2.03	0
TCGA-55-6979	7.90	1
TCGA-78-7150	22.20	1
TCGA-55-6975	3.93	1
TCGA-49-4505	14.27	1
TCGA-MP-A4SY	50.03	1
TCGA-86-8278	15.87	0
TCGA-05-5423	5.03	0
TCGA-05-4427	26.37	0
TCGA-05-4397	24.37	1
TCGA-38-4628	49.73	1
TCGA-73-4668	15.57	0
TCGA-05-4432	25.37	0
TCGA-78-7147	19.53	1
TCGA-49-4506	33.30	1
TCGA-MP-A4T4	87.23	1
TCGA-78-8660	10.70	1
TCGA-62-A46U	68.90	0

TCGA-78-7148	20.87	1
TCGA-55-6984	25.33	1
TCGA-64-5815	7.47	0
TCGA-50-5932	41.17	1
TCGA-55-6982	33.17	1
TCGA-55-6983	94.10	0
TCGA-78-7166	8.60	1
TCGA-73-4676	5.47	0
TCGA-86-8279	16.07	0
TCGA-86-8674	13.50	0
TCGA-86-6562	12.53	1
TCGA-L9-A743	22.13	0
TCGA-MN-A4N1	27.57	0
TCGA-95-A4VN	18.43	0
TCGA-95-8494	2.80	0
TCGA-L9-A7SV	18.83	0
TCGA-49-6742	14.83	0
TCGA-86-7711	21.00	0
TCGA-95-7562	2.90	1
TCGA-MP-A4TI	14.30	1
TCGA-67-6217	14.07	0
TCGA-86-8055	4.13	1
TCGA-95-7567	5.43	0
TCGA-86-8054	24.83	0
TCGA-62-A471	41.53	0
TCGA-05-5425	29.40	0
TCGA-86-8671	27.97	0
TCGA-44-A47F	11.23	0
TCGA-73-4675	1.33	0
TCGA-97-8176	15.60	1
TCGA-49-4507	8.93	1
TCGA-05-4250	4.03	1
TCGA-NJ-A7XG	20.57	0
TCGA-50-5044	20.80	1
TCGA-05-4396	10.10	1
TCGA-38-4632	45.23	1
TCGA-78-7145	27.53	1
TCGA-78-7156	32.53	1
TCGA-05-4434	15.23	1
TCGA-55-8512	20.23	1
TCGA-55-8620	12.50	1
TCGA-49-AAQV	22.57	1
TCGA-91-6830	2.00	0
TCGA-69-8253	14.20	0

TCGA-44-7669	19.13	1
TCGA-55-7914	6.23	1
TCGA-L9-A50W	14.73	1
TCGA-91-A4BD	20.10	0
TCGA-44-6779	16.67	1
TCGA-MP-A4TK	19.40	1
TCGA-50-5068	49.97	1
TCGA-49-AAR3	63.10	0
TCGA-55-8508	20.57	0
TCGA-53-A4EZ	35.70	0
TCGA-49-6744	29.67	0
TCGA-J2-8192	24.63	0
TCGA-55-6712	5.70	1
TCGA-55-7907	11.43	1
TCGA-69-7978	4.47	0
TCGA-55-7227	31.73	1
TCGA-50-6593	11.20	1
TCGA-55-6981	45.97	1
TCGA-44-6774	12.03	0
TCGA-64-1679	56.20	0
TCGA-MP-A4TC	2.47	1
TCGA-86-7714	20.83	1
TCGA-50-5051	15.93	1
TCGA-50-5930	9.40	1
TCGA-78-7220	26.90	1
TCGA-62-8398	14.80	1
TCGA-50-5936	8.57	1
TCGA-35-5375	8.80	0
TCGA-64-5779	16.90	0
TCGA-62-8399	89.87	0
TCGA-05-4384	14.20	0
TCGA-73-4659	23.70	1
TCGA-05-5420	15.23	0
TCGA-55-1594	39.27	0
TCGA-MP-A4T8	5.37	1
TCGA-78-7536	8.13	1
TCGA-62-A46Y	13.80	1
TCGA-78-7146	5.77	1
TCGA-MP-A4TD	10.23	1
TCGA-62-8402	49.93	1
TCGA-44-3396	13.70	0
TCGA-50-6595	6.30	1
TCGA-50-5072	8.33	1
TCGA-64-1677	20.93	1

TCGA-05-4245	24.33	0
TCGA-NJ-A4YI	0.13	1
TCGA-50-5941	18.90	0
TCGA-49-6745	17.40	0
TCGA-97-7554	25.83	0
TCGA-44-5643	13.90	0
TCGA-95-A4VP	20.17	0
TCGA-95-A4VK	21.70	0
TCGA-49-4490	12.83	1
TCGA-86-8359	14.80	1
TCGA-05-5429	9.17	1
TCGA-05-4418	9.13	1
TCGA-99-8025	35.33	0
TCGA-78-7154	19.77	1
TCGA-49-4494	36.03	1
TCGA-50-6594	12.33	1
TCGA-05-4415	3.03	1
TCGA-78-7158	5.97	1
TCGA-62-8394	4.63	1
TCGA-50-5933	79.77	1
TCGA-64-1680	37.53	0
TCGA-78-7160	22.60	0
TCGA-97-8171	18.93	0
TCGA-L9-A5IP	1.93	1
TCGA-49-6761	11.80	0
TCGA-MP-A4T6	59.67	1
TCGA-49-6743	12.30	0
TCGA-55-8505	14.67	0
TCGA-55-7727	3.97	0
TCGA-86-A4P8	14.37	0
TCGA-49-AAR4	29.30	1
TCGA-MP-A4T9	42.17	1
TCGA-91-6848	7.47	0
TCGA-55-6970	15.47	1
TCGA-49-4512	30.17	1
TCGA-91-6849	1.17	0
TCGA-69-7974	6.13	0
TCGA-97-8547	21.90	0
TCGA-55-8615	14.87	0
TCGA-55-7283	20.30	0
TCGA-38-6178	14.93	0

Survival status: 0 represent alive, 1represent dead.
