# Supplementary figures and images for: Expression Status and Prognostic Value of m6A RNA Methylation Regulators in Lung Adenocarcinoma
Source: Life (Basel). 2021 Jun 26;11(7):619. doi: 10.3390/life11070619 (PMC8306618; doi:10.3390/life11070619)

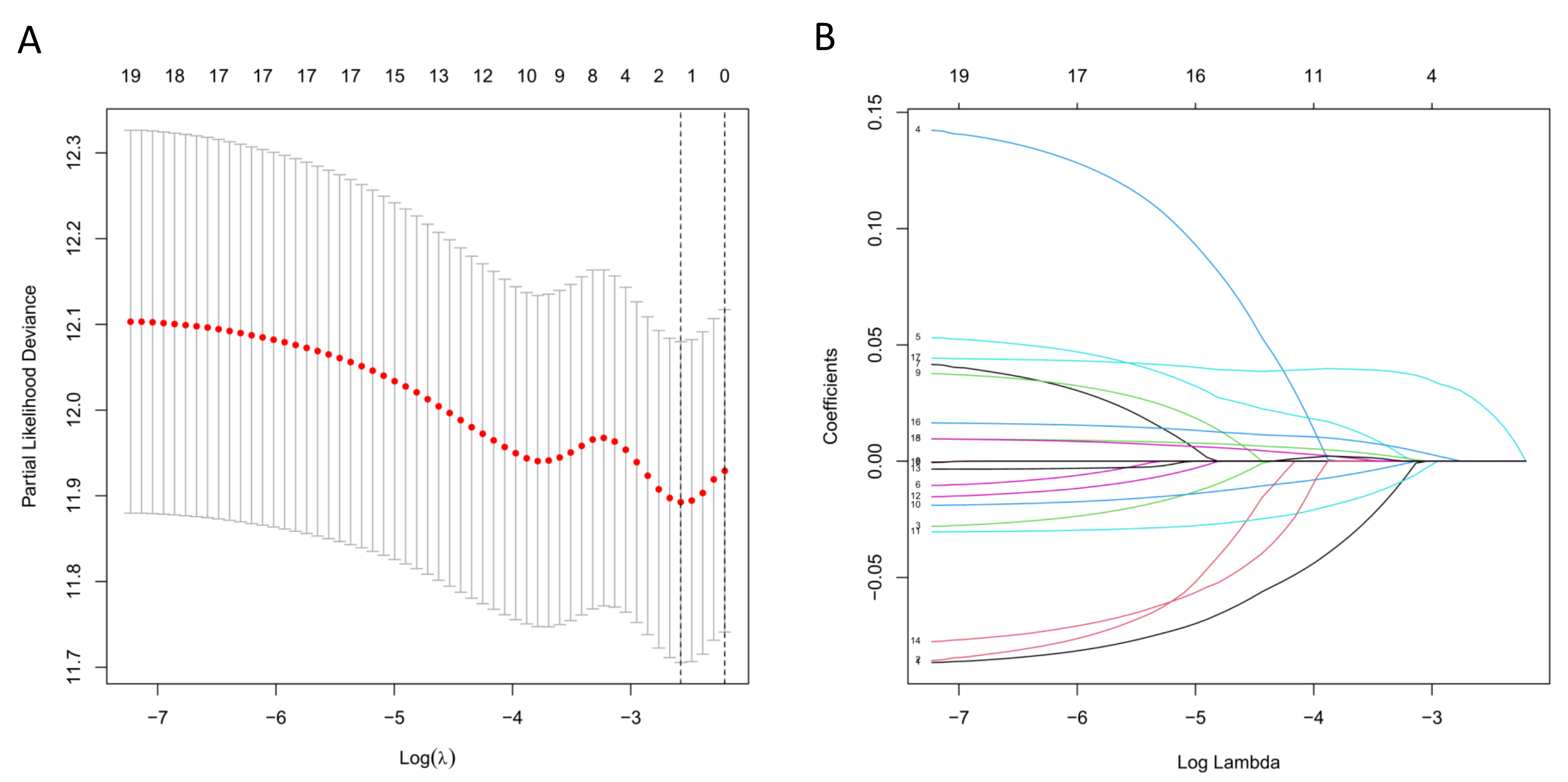

Supplement: Supplementary file 1 [file life-11-00619-s001.zip › life-1174247-supplementary/Supplemental material 2.tif]
